# Supplementary material for: How urban form impacts flooding
Source: Nat Commun. 2024 Aug 19;15:6911. doi: 10.1038/s41467-024-50347-4 (PMC11333580; doi:10.1038/s41467-024-50347-4)
Supplement: Supplementary file 1 — Supplementary Information [file 41467_2024_50347_MOESM1_ESM.pdf]

How Urban Form Impacts Flooding

Sarah K. Balaian[1], Brett F. Sanders[1,2],* and Mohammad Javad Abdolhosseini Qomi[1†]
[1] *Department of Civil and Environmental Engineering, University of California, Irvine, CA 92697, USA.* and
[2] *Department of Urban Planning and Public Policy, University of California Irvine, CA 92697, USA.*
(Dated: June 26, 2024)

I. URBAN FORM DATA

The values for urban porosity and average building side length are calculated for each 1 km x 1km cell (k) within a city using the building footprints. The urban porosity is calculated as $\phi_k = 1 - A_b/A_t$, where A_b is the total area of buildings determined by the building footprint data and A_t is the total area of the cell, *i.e.,* $1000km^2$. Average building side length is simply the average of all building side lengths from the building footprint data. The final averaged values for ϕ and D are presented in Table S1.

The digital elevation map (DEM) [1] is used to determine the maximum slope (α) within each cell and its corresponding direction (θ). This direction is then used to orient the calculation of effective chord length in each cell since we assume the direction of the steepest slope will dictate the dominant flow direction. To calculate the effective chord length, we extend 5,000 lines within the cell in the direction of the steepest slope and we measure the length of chords between buildings. These chord lengths are then used to calculate the effective chord length; see Eq. 5. Averaged values for slope and effective chord length are presented in Table S1.

II. HYBRID REVERSE MONTE CARLO

We generate urban forms through a hybrid reverse Monte Carlo (HRMC) algorithm, which was first introduced to improve upon the modeling of amorphous carbon structures [2, 3]. Using user-defined constraints to inform the placement of buildings and an energy penalty to avoid unrealistic or physically improbable configurations, this probabilistic algorithm is capable of generating unique urban forms that feature accurate geometric attributes.

Here, we employ the Mermin order parameter as the main user-defined constraint. The desired porosity ϕ' and spatial order χ'_{c_n} are defined, and the initial configuration starts as a two-dimensional ordered lattice. It is important to note that the initial point configuration may take any form [4]. The number of points N in the initial configuration is determined by the user-defined ϕ', where each point will signify a building footprint covering a constant area. The initial spatial order $\chi_{c_n,old}$ is calculated for this configuration, which initially equals one for our lattice. One point is arbitrarily chosen and moved in a random direction and distance, and the new spatial parameter $\chi_{c_n,new}$ is calculated. These are both compared to the desired value of χ'_{c_n} and an error G^2 is calculated as follows:

$$\begin{aligned} G^2_{old} &= (\chi_{c_n,old} - \chi'_{c_n})^2 \\ G^2_{new} &= (\chi_{c_n,new} - \chi'_{c_n})^2 \end{aligned} \tag{1}$$

If $G^2_{new} < G^2_{old}$, the new configuration is accepted, it becomes the initial configuration and $\chi_{c_n,old} = \chi_{c_n,new}$. Otherwise, the move is rejected and the old configuration and $\chi_{c_n,old}$ is retained. A new point is moved and the process is then repeated. This process continues until the error value $G^2 < tolerance$ and $\chi_{c_n} = \chi'_{c_n}$.

This algorithm, while robust, lends way to unrealistic configurations in which buildings overlap or are impractically close. Not only does this result in inaccuracies in ϕ and χ_{c_n}, but it also over-complicates the subsequent mesh, reducing overall efficiency and accuracy. To avoid this, we introduce a binary energy penalty in the calculation of G^2 to avoid moves that lead to unrealistic configurations. The energy term (E) is accounted for as follows:

$$G^2 = E + (\chi_{c_n} - \chi'_{c_n})^2 \tag{2}$$

* ORCID: https://orcid.org/0000-0002-1592-5204 & E-mail: bsanders@uci.edu
† ORCID: https://orcid.org/0000-0001-6911-0994 & E-mail: mjaq@uci.edu

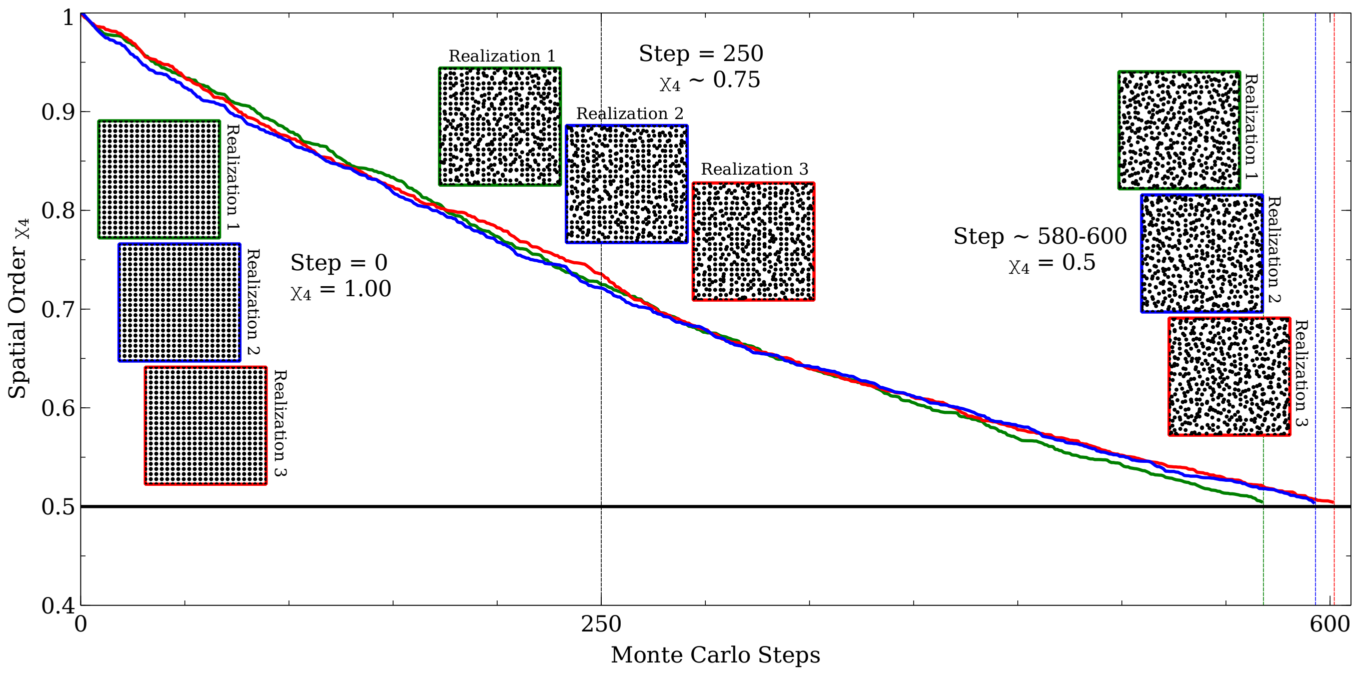

FIG. S1. Three realizations that converge from an initial 2D lattice of points to the final configuration with desired Mermin order value. Each realization maintains a porosity of $\Phi = 0.55$ and converges towards a desired Mermin order value of $\chi_4 = 0.5$. They follow similar paths and reach convergence somewhat simultaneously, but create three different realizations.

Since the energy term is simply a binary to accept or deny a move, the value of the term is arbitrary so long as it ensures $G^2_{new} > G^2_{old}$. The energy term is determined with respect to point j that has been moved and is defined as follows:

$$E = E_j = \sum_{k=1}^{N} E_{j,k} \tag{3}$$

$$E_{j,k} = \begin{cases} 1000 & \frac{d}{D} < \frac{d}{D}_{max} \\ 0 & otherwise \end{cases} \tag{4}$$

where d is the distance between the centroids of building j and each neighboring building k. An analysis of mesh and simulation efficiency determined the minimum acceptable distance between two buildings to be 20 percent of the building side length (D), establishing $(d/D)_{max}$ as 1.2.

As the algorithm iterates through this process, the arrangement and corresponding Mermin order value converges towards the desired final configuration, see Fig. S1. The number of steps taken to reach the desired outcome depends on many factors, such as the number of total buildings in the domain, the desired χ_{c_n}, the tolerance for an acceptable final configuration, and the inherent randomness of the algorithm.

To remove the channel size effects on the asymptotic value of flood depth in drawdown profiles, we needed to extend our channel length to 10 km. Ideally, we want to forgo defining a unit cell and populate the 10 km channel with buildings at a given porosity and order parameter. However, we cannot do this due to the limitations of the Hybrid Reverse Monte Carlo (HRMC) approach. In fact, it is computationally unfeasible to converge HRMC for a 10 km channel. To resolve this computational limitation, we define a unit cell, by repetition of which we could generate our 10 km channels. The size of this unit cell has two constraints: 1) it should be much larger than the building size, and 2) it should be greater than the chord length commonly found in urban forms across the world. As demonstrated in Fig. 5(c), the maximum average chord length in the twenty cities we study belongs to São Paulo, which is ∼12 times the average size of buildings. We also note that the standard deviations are not too large and in the order of a couple of building length. With this length and computational cost in mind, we chose a unit cell which is ∼33 times the size of a building.

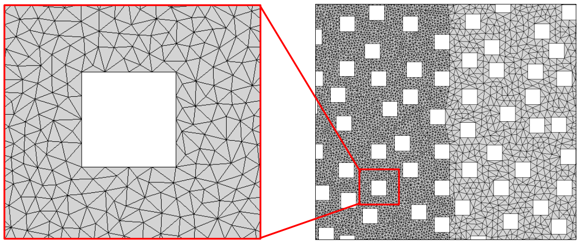

FIG. S2. A mesh that is generated for a specific urban form. The image on the right illustrates the domain at the transition from fine to coarse mesh. The image on the left magnifies the fine mesh. All buildings are represented as holes in the mesh.

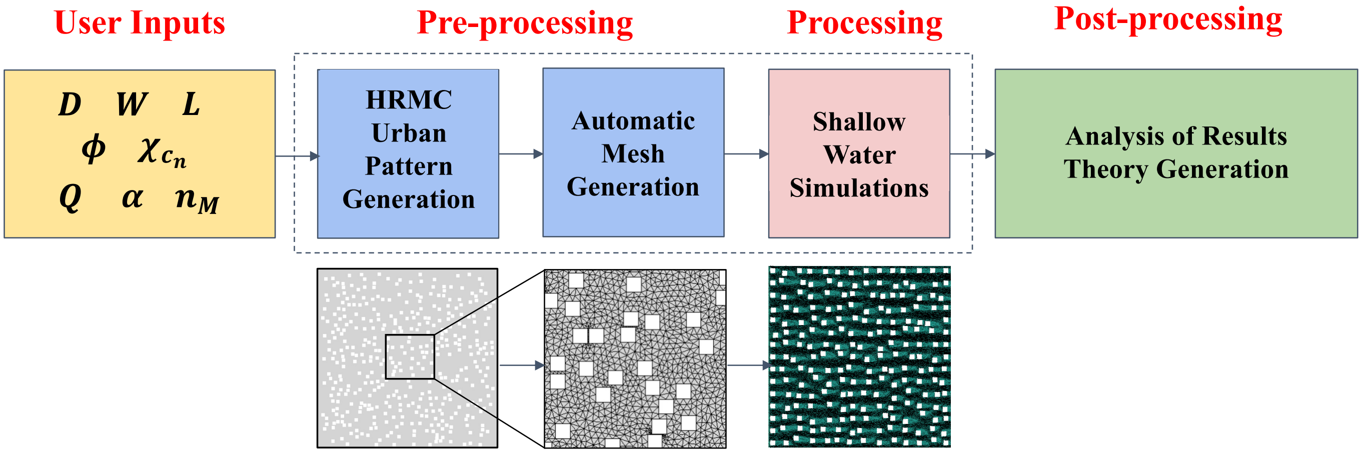

FIG. S3. Framework for the high-throughput computational algorithm which runs thousands of models with differing configurations and input parameters.

III. HIGH-THROUGHPUT COMPUTATIONAL FRAMEWORK

We have developed a framework to integrate the creation of synthetic urban forms, mesh generation, shallow water simulation, and data post-processing. A visual of the algorithm's framework is shown in Fig. S3. Taking inputs of building size D, urban porosity Φ and spatial order χ_{c_n}, channel dimensions L and W, flow rate Q, bottom slope α, and Manning coefficient n_m, the algorithm steps into the HRMC urban pattern generation described in Section II. Once the urban form is generated and populated with buildings, the mesh generation tool Triangle [5] is implemented to produce a triangular mesh constrained by building walls and no-flow boundaries for subsequent simulations; see Fig. S2 for mesh details. Next, flow is simulated using the finite volume model ParBreZo [6]. The output files generated from ParBreZo include spatiotemporal values for flood heights and velocities.

This high-throughput framework allows us to perform parametric studies by generating thousands of models with different building configurations and realizations of urban form, but also different slopes and upstream flow rates. We employ this framework to generate over 1,600 flooding scenarios within our synthetic cities, which facilitates the collection of statistically sufficient data that informs the development of our mean-flow theory. The urban form and flow attributes for each of these scenarios, and the resulting flood heights and velocities, are provided in the dataset 'Supplementary Data 1'.

IV. CHORD LENGTH ANALYSIS

Here, we introduce the normalized effective chord length $\bar{l}_c$, which is the expectation of the normalized chord length. Chord lengths quantify the distance between two neighboring buildings. They are calculated by extending a random point within the domain in any given direction until it intersects a building or channel boundary at each end. The

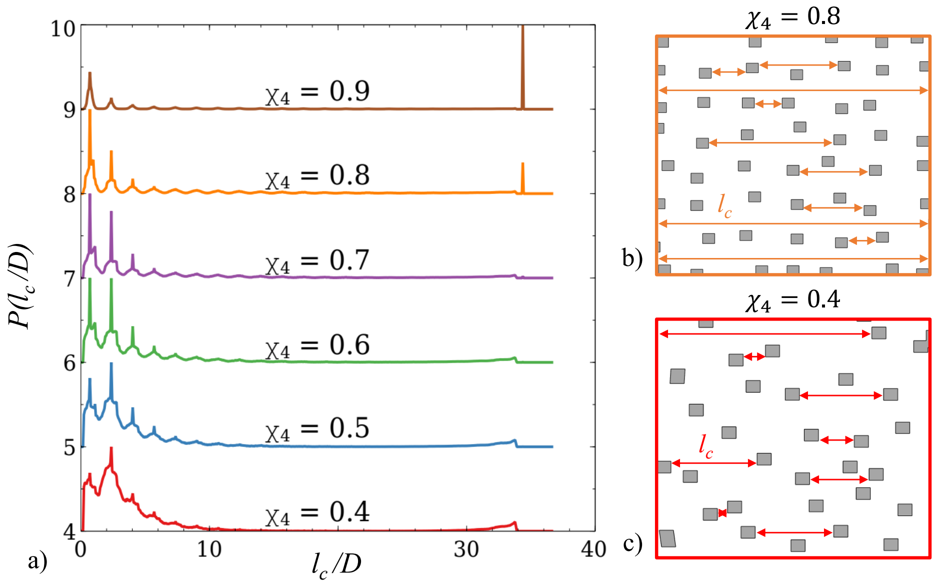

FIG. S4. Determination of chord lengths and corresponding distribution for realizations of $\phi = 0.6$ and $0.4 < \chi_4 < 0.9$ with $\theta = 0$. The distributions have been scaled to range between zero and unity. To the right are schematic representations of chord length determination for realizations of $\chi_4 = 0.6$ and $\chi_4 = 0.8$.

direction can be specified by the chord angle θ with respect to the x-axis, where $0 < \theta < \pi$. For simplicity we only consider the chord length expectation in the direction of steepest slope, which is $\theta = 0$ in our synthetic cities, see Fig. S4. The $\bar{l_c}$ is defined as:

$$\bar{l_c} = \int_3^{l_u/D} l'_c P(l'_c) d(l'_c) \tag{5}$$

where $l'_c = l_c/D$, D is the side length of a building, and l_u is the length of the unit cell. The expectation is only calculated for chord lengths greater than three times D, since shorter chords do not contribute to efficient drainage. To ensure accurate calculation of $\bar{l_c}$, an automated algorithm measures chord lengths along the x-direction, *i.e.*, $\theta = 0$, for 10,000 arbitrary points within the domain.

Once all the chord lengths for a given realization are obtained, we find the distribution of chord lengths ranging from zero to the length of the unit cell. The distributions for realizations with $\phi = 0.6$ can be seen in Fig. S4(a). The added disorder in the system diminishes the longer chords and increases variations in small chords, effectively decreasing flood passage for drainage. This can be visually observed in Fig. S4(b-c); the realization with $\chi_4 = 0.8$ features longer unobstructed flow pathways compared to the realization with $\chi_4 = 0.4$.

V. REDUCING COMPLEXITY OF URBAN FLOODING

A. Dimensional Analysis

Here, we leverage the dimensional analysis technique to reduce the number of independent parameters and identify the dimensionless quantities that drive the flood behavior in the urban environments. To reduce the complexity of urban flood modeling, we use an idealized channel width (W) and length (L), see Fig. S5. The channel bottom slope (α) and Manning's coefficient (n_M) both remain constant throughout the channel. For simplicity, we disperse N square buildings with side length D along the channel, where the centroid of building i is designated via $\mathbf{r}_i$. A fluid with dynamic viscosity (η) and density (ρ) is injected at the volumetric flow rate per width ($q = Q/W$) through the domain's inlet located at the left side of the channel. The fluid experiences gravity (g) and flows in between buildings, experiencing drag coefficient (C_D) and clearing the domain at the dry outflow boundary condition on the right side of the domain. We can express the flood water heights at any point along the channel ($h(x,y)$) as follows:

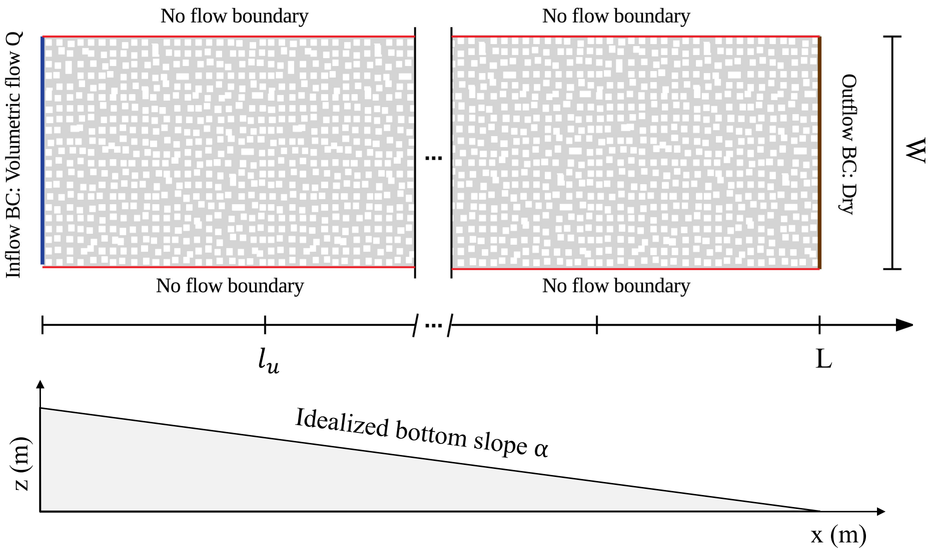

FIG. S5. A schematic representation of the idealized channel domain generated in this work. The dashed line represents the edge of the unit cell with length l_u which is duplicated along the flood channel to populate the entire channel of length L with buildings.

$$h = f(q, \alpha, C_D, n_M, D, W, L, g, \rho, \eta, x, y, \mathbf{r}_1, \mathbf{r}_2, ..., \mathbf{r}_N) \tag{6}$$

If the Buckingham Pi theorem is applied to this description of water heights, we find that the rank of the exponent matrix is three, meaning the number of independent dimensionless quantities is $2N + 10$. This application of dimensionless variables dramatically reduces the representation of water heights to

$$\frac{h^2 gD}{q^2} = f(\alpha, C_D, \frac{g n_M^2 D}{C_D h^{4/3}}, \frac{\rho q D}{h\eta}, \frac{gD^3}{q^2}, \frac{D}{W}, \frac{D}{L}, \frac{x}{D}, \frac{y}{D}, \frac{\mathbf{r}_i}{D}) \tag{7}$$

In the limit of fully developed gravitational flows, *i.e.,* high Reynolds numbers, $\frac{\rho q D}{h\eta}$ becomes significantly large, and the flow becomes independent of the Reynolds number, which is in line with the common assumptions in shallow water modeling. We also note that in the case of dense urban environments, the bottom shear becomes increasingly negligible compared to the drag force. The Manning coefficient is also two orders of magnitude smaller than the drag coefficient. The dimensionless $\frac{g n_M^2 D}{C_D h^{4/3}}$ variable, which can be thought of as the bottom shear-to-drag force ratio, approaches zero. Therefore, this term can be safely eliminated in our analysis. These two assumptions simplify Eq. 7 as follows:

$$\frac{h^2 gD}{q^2} = f(\alpha, C_D, \frac{gD^3}{q^2}, \frac{D}{W}, \frac{D}{L}, \frac{x}{D}, \frac{y}{D}, \frac{\mathbf{r}_i}{D}) \tag{8}$$

It is constructive at this point to look at the relationship between flood height and inlet volumetric flow rate. To this end, we ran shallow water simulations through idealized urban models of varying density and disorder. We characterize the urban density via porosity (ϕ) and disorder via the Mermin order parameter (χ_{c_n}); see the main text for definitions. Regardless of the urban density and disorder, the flood height scales linearly with the inlet volumetric flow rate, *i.e.,* $h \propto q$, see Fig. 3(b) in the main text. This means that the dimensionless variable $\frac{gD^3}{q^2}$ should not play a significant role; otherwise, the relation between h and q becomes nonlinear. This further simplifies Eq. 7 as follows:

$$\frac{h^2 gD}{q^2} = f(\alpha, C_D, \frac{D}{W}, \frac{D}{L}, \frac{x}{D}, \frac{y}{D}, \frac{\mathbf{r}_i}{D}) \tag{9}$$

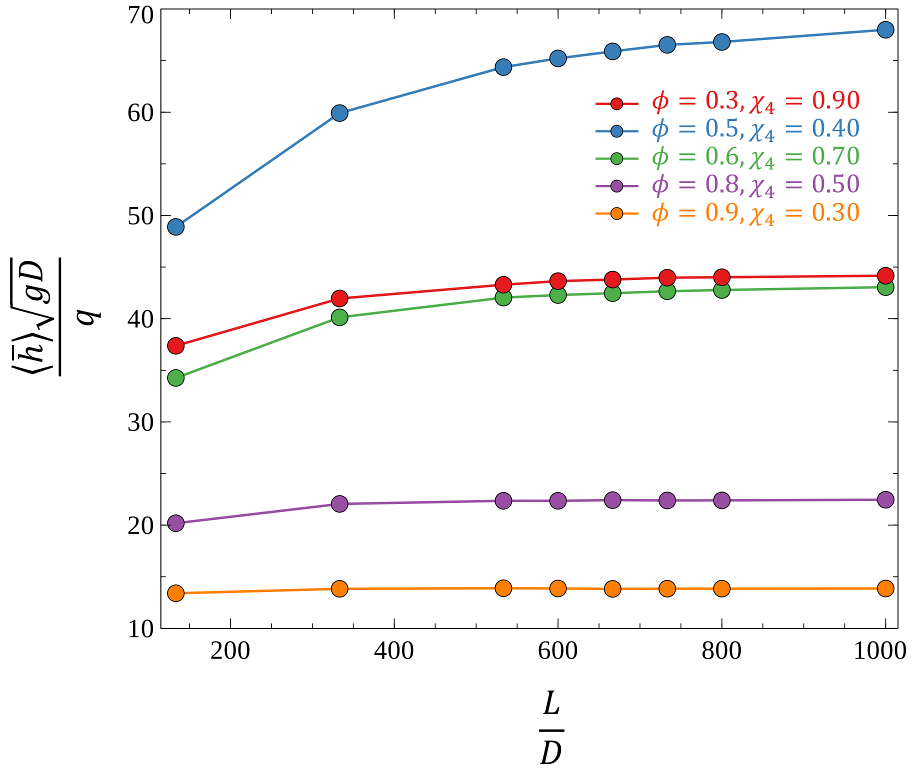

FIG. S6. Exploring the relationship between the inlet flood height (h) and (a) flow rate (Q), (b) normalized channel length (L/D), (c) bottom slope (α), and (d) normalized building side length (D/W). Simulations for multiple realizations of each urban form produce flood heights.

The two dimensionless parameters D/W and D/L signify the impact of the simulation domain's size effect on the flood height. Otherwise, these two parameters signify size effect within a mechanics context. As shown in Fig. S6, the dimensionless flood height plateaus at large L/D. This means that at the limit of very large channels, where D/L goes to zero, this size effect can be safely neglected. Similar conclusions are valid for the other size effect of the channel width. Therefore, Eq. 9 reduces to:

$$\frac{h^2 gD}{q^2} = f(\alpha, C_D, \frac{x}{D}, \frac{y}{D}, \frac{\mathbf{r}_i}{D}) \tag{10}$$

At this point, we perform a parametric study to characterize the impact of the bottom slope on flood height. We find that for a wide range of urban porosity and order, the flood height scales with $\alpha^{-\frac{1}{2}}$, see Fig. 3(c) in the main text. This further simplifies Eq. 10 as follows:

$$\frac{h^2 g\alpha D}{q^2} = f(C_D, \frac{x}{D}, \frac{y}{D}, \frac{\mathbf{r}_i}{D}) \tag{11}$$

The left-hand side of this equation can be considered the ratio of gravitational-to-drag forces if the relation is linear for C_D. Therefore, the above quation simplifies as follows:

$$\frac{h\sqrt{g\alpha D}}{q} = C_D f(\frac{x}{D}, \frac{y}{D}, \frac{\mathbf{r}_i}{D}) \tag{12}$$

B. Averaging Inspired by Statistical Mechanics

With the assistance of dimensional analysis and shallow water modeling, we have so far shown that the dimensionless flood height ($\frac{h\sqrt{g\alpha D}}{q}$) at an arbitrary point (x, y) in the channel is the function of the number of buildings N and

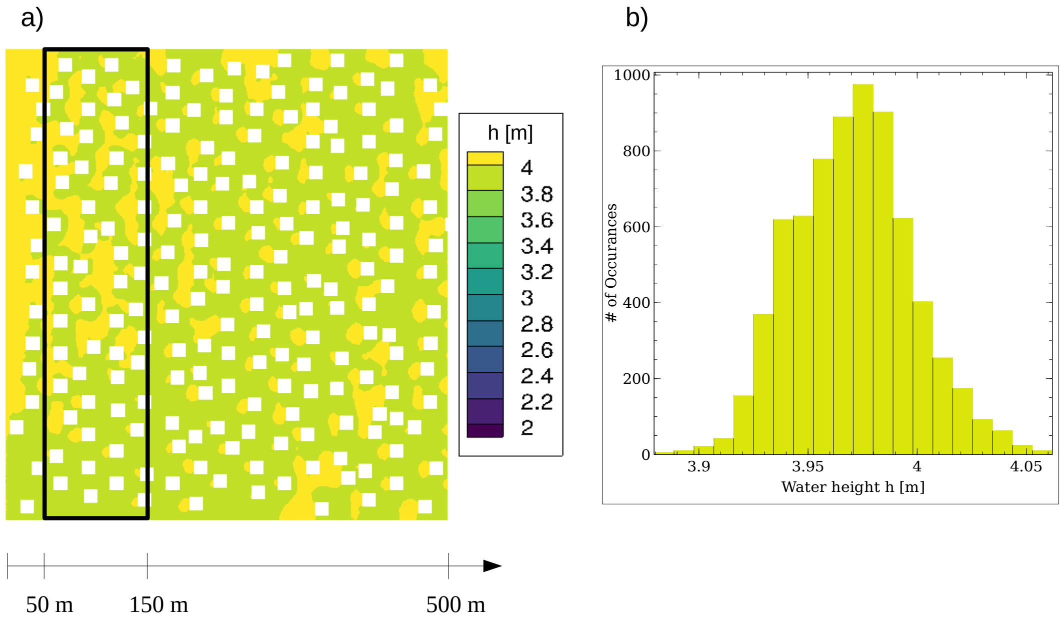

FIG. S7. a) The flood height color map for a unit cell of a long channel. The flood height is not uniform in the rectangular region designated by the thick black line and is b) distributed normally for this particular building distribution pattern.

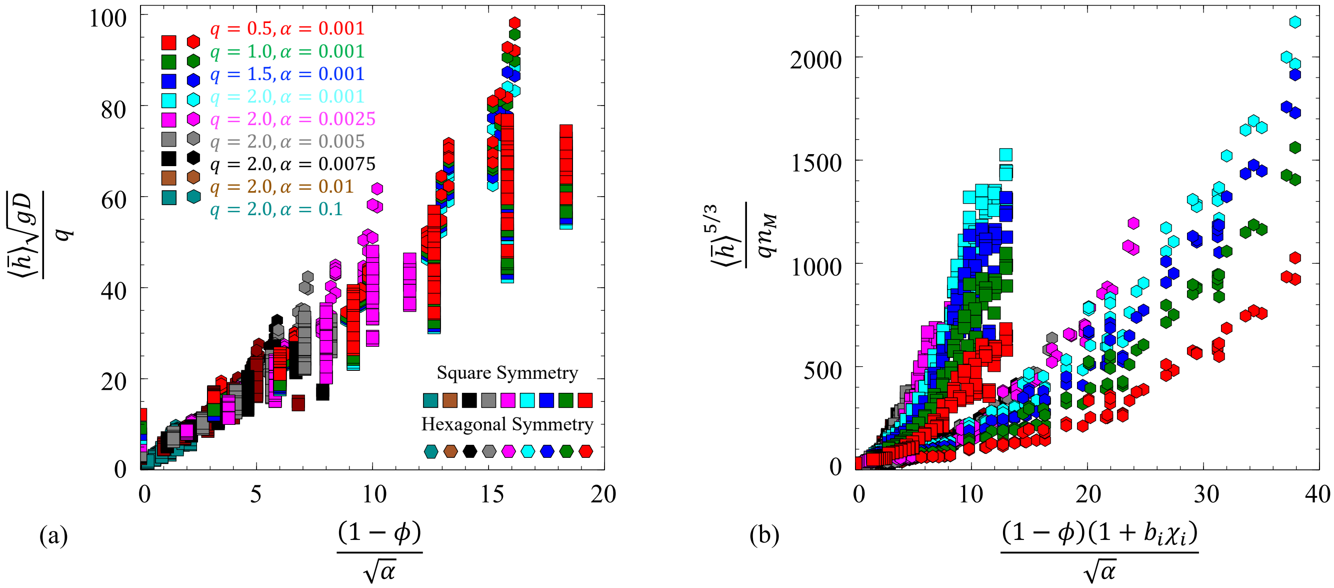

FIG. S8. Visual representation of the data collapse (a) of dimensionless inundation when only considering porosity to describe the urban texture and (b) considering $\langle \bar{h} \rangle^{5/3}/(q n_M)$ as the dimensionless inundation that is valid only at high urban porosity.

their location $\mathbf{r}_i$. When viewed in a statistical sense, the flood height at a point might not matter as much as the average flood height in a region within the floodplain. As shown for a specific example of building arrangement in Fig. S7, the flood height can be treated as a statistical distribution for which the statistical moments carry significant information regarding the flooding behavior. In this spirit, the first moment, average, of flood height can be written as:

$$\frac{h\sqrt{g\alpha D}}{q} = C_D\langle f(\frac{x}{D},\frac{y}{D},\frac{\mathbf{r}_i}{D})\rangle = \frac{C_D}{A}\int_{\Omega_0} f(\frac{x}{D},\frac{y}{D},\frac{\mathbf{r}_i}{D})dA \tag{13}$$

where the integral averaging is performed over a domain Ω_0 with the area A. According to the mean value theorem for definite integrals, we can simplify the integral as follows:

$$\frac{h\sqrt{g\alpha D}}{q} = C_D f(\frac{x_0}{D},\frac{y_0}{D},\frac{\mathbf{r}_i}{D}) \tag{14}$$

where (x_0, y_0) is a point within domain Ω_0.

If we are not particularly interested in a specific configuration of buildings in the flood channel $\mathbf{r}_i$, then Eq. 14 can be averaged akin to the ensemble averaging in statistical physics of particles,

$$\frac{\langle\bar{h}\rangle\sqrt{g\alpha D}}{q} = \frac{\langle C_D\rangle}{Z}\int_{\Omega_{\mathbf{r}_i}} p(\mathbf{r}_i) f(\frac{x_0}{D},\frac{y_0}{D},\frac{\mathbf{r}_i}{D})d\mathbf{r}_i \tag{15}$$

where $\langle\bar{h}\rangle$ denotes the ensemble average of the flood height in a given domain and the averaging is performed over the phase space of all building configurations, $\Omega_{\mathbf{r}_i}$, in the flood channel. We note that the phase space is an abstract $2N$ dimensional space. The probability density function $p(\mathbf{r}_i)$ designates the possibility of a certain configuration in the phase space. For instance, the probability can be defined in such a way that possibility of buildings overlapping each other is zero. The Z variable represents the so-called partition function defined as:

$$Z = \int_{\Omega_{\mathbf{r}_i}} p(\mathbf{r}_i)d\mathbf{r}_i \tag{16}$$

Eq. 15 is remarkable in that it places urban flood modeling on par with thermodynamics, in which the macroscopic properties of the system are the average of microscopic states. Otherwise said, the ensemble-averaged dimensionless flood height behaves akin to thermodynamic state functions. It is well-established in classical thermodynamics that state functions are a function of state variables of the system, which are related to the average textural attributes of the system at the microscale. This motivates us to express the complex average in Eq. 15 in terms of state variables commonly used in studying the thermodynamics of disordered porous media, such as porosity (ϕ), disorder (χ_{c_n}), and effective chord length ($\bar{l_c}$):

$$\frac{\langle\bar{h}\rangle\sqrt{gD}}{q} = \frac{\langle C_D\rangle}{\sqrt{\alpha}} G(\phi, \chi_{c_n}, \bar{l_c}, ...) \tag{17}$$

where G is an unknown function to be determined via numerical shallow water modeling or experimentation. We also note that the function can depend on one, two, or more state variables and might not necessarily be unique. This is confirmed through the determination of G; as seen in Fig. S8(a), the packing fraction collapses data only at high porosity and becomes increasingly insufficient in dense textures, where the disorder becomes a controlling factor.

VI. GLOBAL URBAN FLOOD HAZARD ANALYSIS

Urban form geometric attributes are used to determine non-dimensionalized flood depths (H^*) and intensities (P^*) for cities globally as follows:

$$H^* = \frac{\langle\bar{h}\rangle}{h_{ref}} = \frac{\frac{q\beta}{\sqrt{gD}}}{(\frac{q_{ref}n_m}{\sqrt{\alpha}})^{3/5}} = \frac{q\beta}{\sqrt{D}}\left(\frac{\alpha_{ref}^{3/10}}{q_{ref}^{3/5}n_m^{3/5}\sqrt{g}}\right) \tag{18}$$

$$P^* = \frac{\langle\bar{hu}\rangle}{hu_{ref}} = \frac{q}{\phi}\frac{1}{q_{ref}} \tag{19}$$

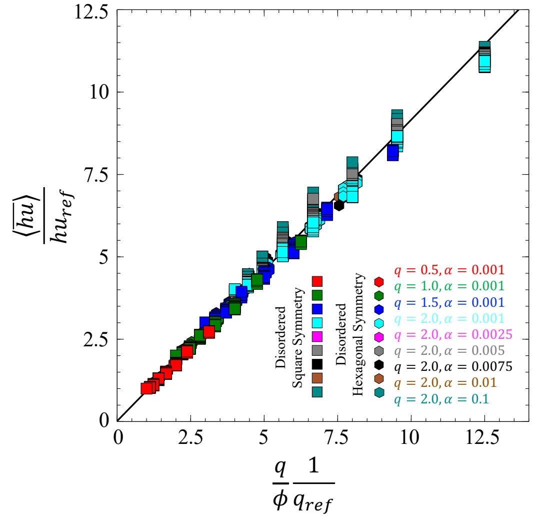

FIG. S9. The linear relationship between non-dimensionalized flood intensity $P^* = \langle \bar{hu} \rangle / hu_{ref}$ and the inverse of porosity for our synthetic cities. The color of each data point represents a different combination of slope and flow rate. The marker represents the underlying symmetry of the configuration.

where h_{ref} is the flood height of an obstruction-less channel with $q_{ref} = 0.001\ m^3/s$ and $\alpha_{ref} = 0.1$. As presented in Fig. S9, flow simulations featuring the same flow rate q show that the flood intensity is governed by porosity at the urban scale and is independent of order.

We obtain information on extreme events within our twenty cities dating back to the year 2000 to establish a correlation between our dimensionless parameters and monetary damages that accrue during flood disasters. We do not consider flooding events that occurred before the year 2000 for a few reasons. Mainly, satellite data for determining precipitation rates is limited to the past 20 years. Also, due to the ever-evolving nature of urban areas, flood attributes from past decades may not be representative of the city's current urban form. The flood data is extracted from the Emergency Events Database, (EM-DAT), a disaster database distributed in open access by the Centre for Research on the Epidemiology of Disasters (CRED)[7]. From this database, we extracted event start and end dates and monetary damage values. These damage values are normalized by the respective country's gross domestic product (GDP), which is obtained from the World Bank database [8]. Information pertaining to the flood events considered in this study is presented in Table S2. The H^* and P^* values for each extreme event are determined using a flow rate estimated by the rational method using the maximum precipitation rate during the entire length of the respective flooding event, and assuming no infiltration/losses. Precipitation data is obtained from the PERSIANN (Precipitation Estimation from Remotely Sensed Information using Artificial Neural Networks) system, developed by the Center for Hydrometeorology and Remote Sensing (CHRS) at the University of California, Irvine (UCI)[9].

---

[1] D. Yamazaki *et al.*, A high-accuracy map of global terrain elevations, Geophysical Research Letters **44**, 5844 (2017).
[2] G. Opletal *et al.*, Hybrid approach for generating realistic amorphous carbon structure using metropolis and reverse Monte Carlo, Molecular Simulation **28**, 927 (2002).
[3] C. Bousige, A. Boţan, F.-J. Ulm, R. J.-M. Pellenq, and B. Coasne, Optimized molecular reconstruction procedure combining hybrid reverse Monte Carlo and molecular dynamics, The Journal of Chemical Physics **142**, 114112 (2015).
[4] R. L. McGreevy, Reverse Monte Carlo modelling, Journal of Physics: Condensed Matter **13**, R877 (2001).
[5] J. R. Shewchuk, Triangle: Engineering a 2D quality mesh generator and Delaunay triangulator, in *Applied Computational Geometry Towards Geometric Engineering*, Lecture Notes in Computer Science, edited by M. C. Lin and D. Manocha (Springer, Berlin, Heidelberg, 1996) pp. 203–222.
[6] B. F. Sanders, J. E. Schubert, and R. L. Detwiler, ParBreZo: A parallel, unstructured grid, Godunov-type, shallow-water code for high-resolution flood inundation modeling at the regional scale, Advances in Water Resources **33**, 1456 (2010).
[7] D. G. Sapir and C. Misson, The Development of a Database on Disasters, Disasters **16**, 74 (1992).
[8] W. Bank, GDP (current US$) (2021).
[9] P. Nguyen *et al.*, The CHRS Data Portal, an easily accessible public repository for PERSIANN global satellite precipitation data, Scientific Data **6**, 180296 (2019).

TABLE S1. City-averaged building size (D), bottom slope (α), urban porosity (ϕ), effective chord length ($\bar{l_c}$), and non-dimensionalized flood inundation (H^*) and flood intensity (P^*) for twenty cities worldwide. The 90% confidence interval for all 1 km x 1 km cells in each city is presented. †Building footprint data for this city may not be sufficient.

City-Averaged Urban Data						
City	D	α	Φ	$\bar{l_c}$	H^*	P^*
1. Los Angeles, CA, USA	11.87 ± 0.19	0.11 ± 0.01	0.78 ± 0.00	7.92 ± 0.20	0.13 ± 0.01	1.29 ± 0.01
2. Seattle, WA, USA	10.69 ± 0.35	0.19 ± 0.01	0.82 ± 0.01	9.26 ± 0.40	0.06 ± 0.00	1.23 ± 0.01
3. Miami, FL, USA	12.48 ± 0.37	0.02 ± 0.01	0.79 ± 0.01	7.90 ± 0.58	0.26 ± 0.02	1.26 ± 0.01
4. Boston, MA, USA	12.91 ± 0.64	0.10 ± 0.01	0.80 ± 0.01	7.42 ± 0.51	0.09 ± 0.01	1.27 ± 0.02
5. San Francisco, CA, USA	12.87 ± 0.53	0.20 ± 0.02	0.70 ± 0.01	6.50 ± 0.68	0.09 ± 0.01	1.44 ± 0.03
6. New Orleans, LA, USA	13.00 ± 0.40	0.02 ± 0.00	0.78 ± 0.01	8.28 ± 0.60	0.22 ± 0.01	1.29 ± 0.02
7. New York, NY, USA	15.84 ± 0.45	0.07 ± 0.00	0.75 ± 0.01	6.28 ± 0.28	0.14 ± 0.01	1.37 ± 0.02
8. Chicago, IL, USA	11.42 ± 0.25	0.02 ± 0.00	0.77 ± 0.00	8.47 ± 0.34	0.29 ± 0.01	1.31 ± 0.01
9. Virginia Beach, VA, USA	12.00 ± 0.36	0.02 ± 0.00	0.86 ± 0.00	11.19 ± 0.46	0.15 ± 0.01	1.17 ± 0.01
10. Savannah, GA, USA	13.156 ± 0.88	0.03 ± 0.00	0.85 ± 0.01	9.69 ± 0.81	0.13 ± 0.01	1.18 ± 0.01
11. Tampa, FL, USA	12.74 ± 0.41	0.0300 ± 0.00	0.85 ± 0.00	10.22 ± 0.54	0.14 ± 0.01	1.19 ± 0.01
12. London, England	9.45 ± 0.31	0.07 ± 0.00	0.77 ± 0.01	10.99 ± 0.56	0.13 ± 0.01	1.32 ± 0.02
13. Tokyo, Japan	8.36 ± 0.26	0.07 ± 0.00	0.69 ± 0.01	8.10 ± 0.41	0.18 ± 0.01	1.47 ± 0.02
14. Houston, TX, USA	13.46 ± 0.33	0.01 ± 0.00	0.82 ± 0.00	9.92 ± 0.36	0.24 ± 0.01	1.23 ± 0.01
15. Karachi, Pakistan†	15.12 ± 0.68	0.04 ± 0.01	0.71 ± 0.02	6.70 ± 0.82	0.17 ± 0.02	1.45 ± 0.06
16. Mumbai, India†	13.96 ± 0.55	0.07 ± 0.01	0.84 ± 0.00	11.03 ± 0.55	0.10 ± 0.00	1.20 ± 0.01
17. São Paulo, Brazil	5.77 ± 0.13	0.12 ± 0.00	0.59 ± 0.01	12.10 ± 0.55	0.16 ± 0.01	1.75 ± 0.02
18. Jakarta, Indonesia	10.32 ± 0.16	0.03 ± 0.00	0.68 ± 0.01	7.44 ± 0.27	0.28 ± 0.01	1.51 ± 0.01
19. Vancouver, Cañada	10.40 ± 0.39	0.11 ± 0.01	0.77 ± 0.01	8.67 ± 0.65	0.10 ± 0.01	1.31 ± 0.02
20. Lagos, Nigeria	9.54 ± 0.06	0.04 ± 0.00	0.69 ± 0.01	6.51 ± 0.24	0.24 ± 0.01	1.47 ± 0.02

TABLE S2. Flash flooding events considered in this study. This table contains information on flood location, start and end date, monetary damage values, and the respective country GDP. Sufficient monetary damage data is only available for five flash flooding events.

City-Averaged Urban Data						
City	Start Date	End Date	H^*_{flood}	P^*_{flood}	Damages, Adj. (2021 US $)	GDP (2021 US $)
1. Los Angeles, CA, USA	2003/11/12	2003/11/13	0.045	0.444	N/A	2.332e+13
2. Los Angeles, CA, USA	2014/12/02	2014/12/05	0.094	0.920	N/A	2.332e+13
3. San Francisco, CA, USA	2014/12/02	2014/12/05	0.073	1.209	N/A	2.332e+13
4. Houston, TX, USA	2016/04/16	2016/04/19	1.681	8.525	2.439e+09	2.332e+13
5. Karachi, Pakistan	2007/08/10	2007/08/13	0.782	6.605	N/A	3.483e+11
6. Karachi, Pakistan	2017/06/26	2017/09/11	0.754	6.368	N/A	3.483e+11
7. Mumbai, India	2007/06/22	2007/07/04	0.242	3.042	N/A	3.176e+12
8. Mumbai, India	2017/08/29	2017/08/29	0.098	1.227	N/A	3.176e+12
9. São Paulo, Brazil	2007/01/01	2007/01/12	0.192	2.125	N/A	1.609e+12
10. São Paulo, Brazil	2014/01/12	2014/01/13	0.502	5.544	N/A	1.609e+12
11. São Paulo, Brazil	2016/03/10	2016/03/11	0.412	4.555	1.219e+08	1.609e+12
12. Jakarta, Indonesia	2001/02/04	2001/02/18	0.609	3.219	1.653e+07	1.186e+12
13. Jakarta, Indonesia	2017/05/12	2017/05/15	0.151	0.796	2.388e+06	1.186e+12
14. Jakarta, Indonesia	2019/12/31	2020/01/03	0.711	3.756	N/A	1.186e+12
15. Jakarta, Indonesia	2022/10/04	2022/10/09	0.659	3.481	N/A	1.186e+12
16. Lagos, Nigeria	2000/09/20	2000/09/21	0.645	4.030	8.166e+06	4.408e+11
