# Supplementary material for: How urban form impacts flooding
Source: Nat Commun. 2024 Aug 19;15:6911. doi: 10.1038/s41467-024-50347-4 (PMC11333580; doi:10.1038/s41467-024-50347-4)
Supplement: Supplementary file 3 — Description of Additional Supplementary Files [file 41467_2024_50347_MOESM3_ESM.pdf]

Description of Additional Supplementary Files

File Name: Supplementary Data 1
Description: Porosity $\phi * 100$; Mermin order parameter $\chi_{C_n} * 100$; Volumetric flow rate Q; Bottom slope α; Effective nondimensionalized chord length $\overline{l_c}$; Flood intensity at inlet $h * u$; Average flood depth at inlet h; Average flood velocity at inlet u
